# Combination of Poly(lactic) Acid and Starch for Biodegradable Food Packaging

**DOI:** 10.3390/ma10080952

**Published:** 2017-08-15

**Authors:** Justine Muller, Chelo González-Martínez, Amparo Chiralt

**Affiliations:** Universidad Politécnica de Valencia, IIAD, Camino de Vera, s/n, 46022 València, Spain; justinemuller@hotmail.fr (J.M.); cgonza@tal.upv.es (C.G.-M.)

**Keywords:** Poly(lactic) acid, starch, films, blends, multilayer, food packaging, mechanical properties, barrier properties

## Abstract

The massive use of synthetic plastics, in particular in the food packaging area, has a great environmental impact, and alternative more ecologic materials are being required. Poly(lactic) acid (PLA) and starch have been extensively studied as potential replacements for non-degradable petrochemical polymers on the basis of their availability, adequate food contact properties and competitive cost. Nevertheless, both polymers exhibit some drawbacks for packaging uses and need to be adapted to the food packaging requirements. Starch, in particular, is very water sensitive and its film properties are heavily dependent on the moisture content, exhibiting relatively low mechanical resistance. PLA films are very brittle and offer low resistance to oxygen permeation. Their combination as blend or multilayer films could provide properties that are more adequate for packaging purposes on the basis of their complementary characteristics. The main characteristics of PLA and starch in terms of not only the barrier and mechanical properties of their films but also of their combinations, by using blending or multilayer strategies, have been analyzed, identifying components or processes that favor the polymer compatibility and the good performance of the combined materials. The properties of some blends/combinations have been discussed in comparison with those of pure polymer films.

## 1. Introduction

Over the last few decades, the environment has become one of the major global concerns, especially in the face of pollution, the depletion of natural resources and environmental degradation. As a consequence, research efforts have been developed at different levels to find alternative solutions. A specific concern is the field of packaging, which produces great amounts of non-degradable plastic waste accumulated in critical areas around the planet, causing severe problems and representing high recycling costs. Europe ranks second, along with North America, in the global production of plastic materials with 18.5% of the worldwide annual production (322 million tons in total in 2015). In Europe, the packaging sector is the largest one in the plastics industry, since it represents 40% of the total plastic demand, which rises to 49 million tons [1]. 

**Figure 1:**
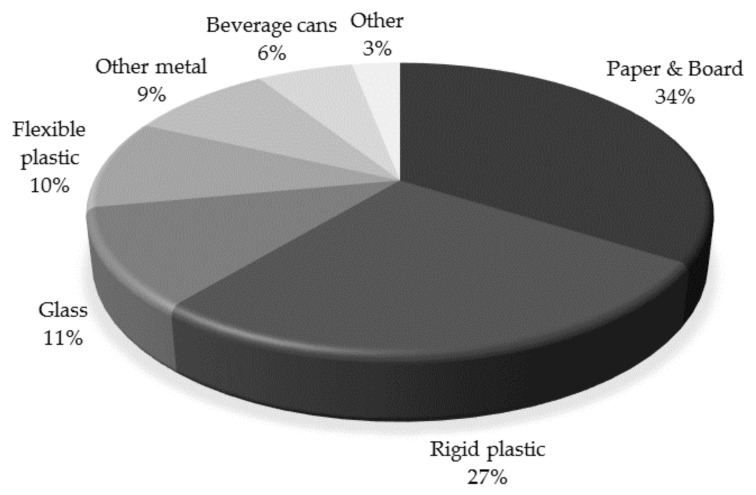
Market share of food packaging materials. Adapted from [2], with permission from publisher.

Food packaging represents an important consumption of different materials, of which plastics have increased exponentially over the past two decades, with an annual growth of approximately 5%. Indeed, plastics represent the second most widely used material for food packaging applications, after paper and cardboard. Figure 1 shows the market share of food packaging materials [2]. Of the plastic materials, petroleum-based plastics, such as polyethylene (PE), polypropylene (PP), polyamide (PA), are widely used for packaging purposes. Despite the environmental issues, plastics are very successful in the packaging market, due to their great combination of flexibility (from film to rigid applications), strength, transparency, stability, permeability and ease of sterilization, all of which making them suitable for food packaging. However, despite their good properties, their use and accumulation imply serious environmental problems and dependence on fossil fuels. 63% of the current plastic waste comes from packaging applications, and it is estimated that less than 14% are recyclable. In 2014, more than 7.5 tons of plastic waste were collected for recycling in Europe [1]. 

To circumvent the growing plastic production and waste and thus the pollution problem, research has been focused on the development of alternative bio-packaging materials, derived from renewable sources, which are biodegradable or compostable. Biopolymers can be used to substitute non-biodegradable plastics with other more natural and eco-friendly materials, named bioplastics, reducing the environmental impact and oil-dependence. Bioplastics are divided into three main categories, on the basis of their origin and biodegradable nature: biobased-non-biodegradable bioplastics (e.g., polyethylene terephthalate (PET), PA), biobased-biodegradable bioplastics (e.g., PLA, polyhydroxyalkanoates (PHA) or starch, other polysaccharides or proteins) or fossil-based biodegradable bioplastics (e.g., polycaprolactone (PCL)) [3]. Therefore, biopolymers are biodegradable, biobased or both and can be classified as those directly obtained from biomass (PS and proteins), synthetic biopolymers from biomass or petrochemicals (e.g., PLA, PCL) or those obtained by microbial fermentation (e.g., PHA). The former are directly extracted from biological and natural resources and they are hydrophilic and somewhat crystalline in nature, making an excellent gas barrier. Biodegradable polyesters (synthetic or biosynthesized) are more hydrophobic and constitute better barriers to water vapor. In general, the functional properties of biopolymer-based materials in terms of their mechanical and barrier properties need to be adapted to food requirements, by using different strategies, such as physical or chemical modifications (crosslinking) or blending with other components, plasticizers or compatibilizers. As a consequence, many studies have been carried out in order to obtain bioplastics with comparable functionalities to those of petrochemical polymers, in order to respond to the environmental problems. 

Poly(lactic) acid and starch have been extensively studied as potential replacers of non-degradable petrochemical polymers on the basis of their availability, adequate food contact properties and competitive cost. Both PLA and starch are semicrystalline polymers, and the crystallinity degree of their films depends both on the source and processing conditions. Crystallinity degree greatly affects the mechanical performance of the material. This review is devoted to analyzing the main characteristics of PLA and starch in terms of not only the barrier and mechanical properties of their films but also of their combinations, by using blending or multilayer strategies, have been analyzed, identifying components or processes that favor the polymer compatibility and the good performance of the combined materials. The properties of some blends/combinations will be discussed in comparison with those of pure polymer films.

## 2. The Main Characteristics of PLA Materials

**Figure 2:**
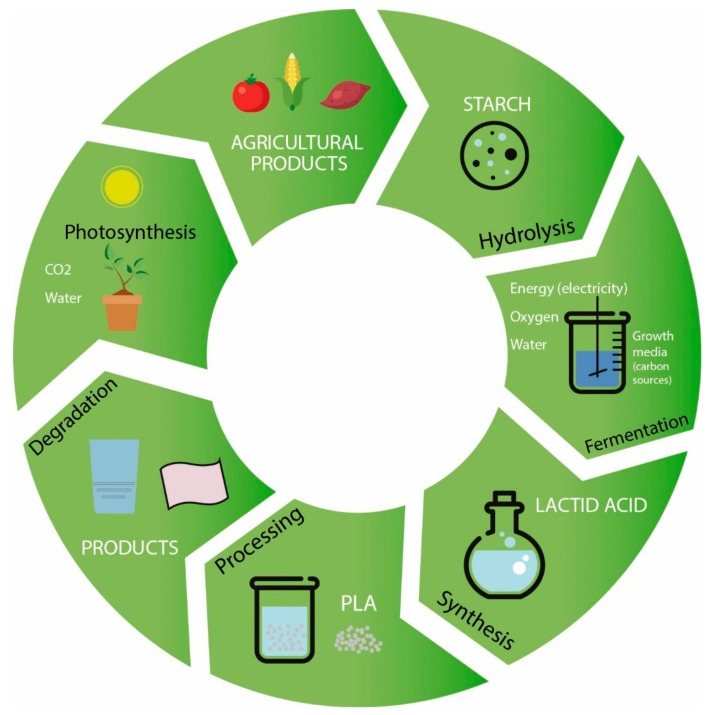
PLA cycle in nature. Adapted from Auras et al. [6], with permission from publisher.

**Figure 3:**
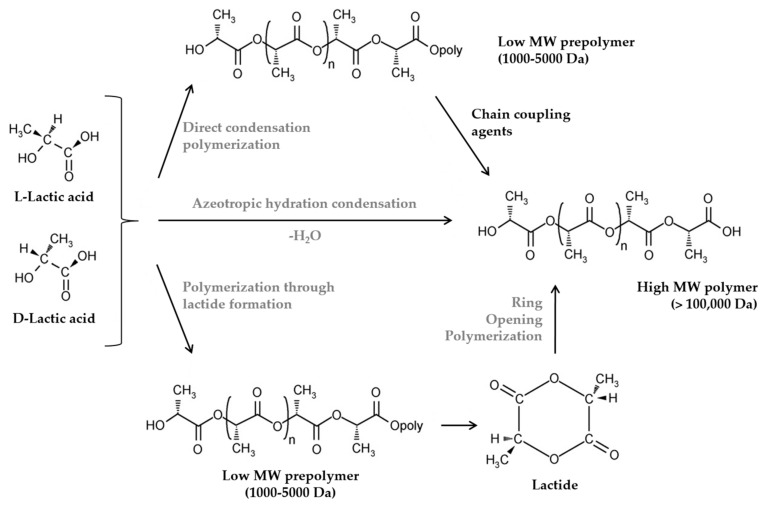
Different routes to produce PLA. Adapted from Auras et al. [6], with permission from publisher.

PLA is a linear aliphatic thermoplastic polyester derived from lactic acid, which is obtained from the fermentation of 100% renewable and biodegradable plant sources, such as corn or rice starches and sugar feed stocks. It can be produced by chemical conversion of corn or other carbohydrate sources into dextrose. Dextrose is fermented to lactic acid followed by polycondensation of lactic acid monomers or lactide. However, the most common way to produce PLA is the Ring Opening Polymerization (ROP) of lactide monomer formed from lactic acid [4]. Figure 2 shows the natural cycle of PLA while Figure 3 presents the different ways to produce PLA. It was reported that the production of PLA resin pellets consumes from 25 to 55% less fossil energy than petroleum-based polymers [5].

Three different stereochemical forms exist for lactide: either L-, D- or both L, D-Lactide (meso-lactide), each one having their own melting properties. PLA is insoluble in water, ethanol, methanol and aliphatic hydrocarbons but it is soluble in chloroform, hot benzene, acetonitrile, which are toxic, but also in acetone, ethyl acetate and dichloromethane. Its degradation half-life goes from six months to two years, depending on its stereochemistry and molecular weight [4]. PLA exhibits many advantages; it is biodegradable, renewable and biocompatible and it has been approved by the Food and Drug Administration (FDA) for direct contact with biological fluids. It is also highly transparent and has good water vapor barrier properties [7,8], comparable to those of petroleum-based plastics, such as polyethylene terephthalate (PET) or polystyrene (PS). Because of the emerging industrial production technologies, PLA is now very competitive in price. Nonetheless, PLA has limited gas barrier capacity due to its hydrophobic nature. Despite its strong resistance (tensile strength from 17 to 74 MPa) [9,10], it is very brittle, with less than 10% of elongation at break [11,12]. 

Due to its good thermal processability, PLA can be tailor-made for different fabrication processes, either injection molding, sheet extrusion, blow molding, thermoforming, film forming, or fiber spinning. However, depending on the process, some parameters must be controlled (D-isomer content, molecular weight distribution). L-, D- or meso-lactide stereoisomers can be incorporated into the polymer backbone, producing different PLA materials for specific applications. Although PLA is hydrophobic, the pellets must usually be dried from 60 to 100 °C for several hours prior to processing to prevent excessive hydrolysis and modifications of the physical properties of the polymer [13]. 

The most common technique used to process PLA is extrusion, which permits the pellets to be mixed homogeneously under high temperature [13]. PLA can also be dissolved in chloroform [9,14,15,16] or other solvents, such as dichloromethane [17], methylene chloride or acetonitrile [18], and cast to obtain films with high transparency and gloss. Because of its initially high cost and its bio-absorption characteristics, PLA was mainly studied for biomedical uses, such as tissue engineering [4]. Nonetheless, the new PLA production techniques and polymerization routes now permit a reduction in the cost of obtaining high molecular weight PLA. As a consequence, PLA is now more readily available for packaging applications and consumer goods. With comparable mechanical and optical properties to those of conventional plastics, PLA could possibly substitute LDPE and HDPE, PS or PET in several food packaging applications. Nevertheless, some factors limit them and different polymer modifications have been carried out to adapt PLA properties to the packaging requirements. 

Table 1 shows the values of tensile properties of neat PLA films reported by different authors. A relatively wide range of tensile strength (14–70 MPa) and deformation at break (1–8%) can be found depending on the type of PLA and process. Since PLA-based materials are rigid and brittle, plasticizers have been added to enhance the mechanical performance of the PLA films. In this sense, many studies have been performed with different plasticizing agents, such as glycerol [19] acetyl tri-n-butyl citrate (ATBC) [20,21], glycerol triacetate (TA) and tributyl citrate (TBC) [22], triacetine tributyl citrate, acetyl tributyl citrate, triethyl citrate and acetyl triethyl citrate [23] or epoxized palm (EPO) and soybean (ESO) oils [24]. Several authors also reported the PLA plasticization with polyethylene glycol (PEG) of different molecular weights: 200 [25], 300 [22], 400 [18], 550 [26], 1000 [10,27], 1500 and 10,000 g·mol^−1^ [20]. As plasticizers decreased the glass-transition temperature (T_g_), a lower stress at yield and a higher elongation at break were observed in the different studies. 

As concerns the barrier properties of PLA films, water vapor permeability (WVP) values (ranging from 1 × 10^−14^ to 4 × 10^−14^ kg·m/s·m·Pa) [7,8,28,29] are low compared to starch films but oxygen permeability is very high (2.4 × 10^−15^ kg·m/s·m·Pa) [30]. As gas permeability is a problem for PLA, Rocca-Smith et al. [31] applied a corona treatment to PLA films and observed improved gas (He, O_2_, CO_2_) barrier properties, with modifications in both the surface and bulk of the films. In particular, the surface analysis revealed an increase in polarity and roughness, while some modifications of the mechanical properties and degree of crystallinity were also observed in the resulting films. 

Another option considered for the purposes of improving PLA properties is to combine it with other biopolymers by using an adequate compatibilization strategy. Different PLA blends have been studied, such as PLA-PHB/cellulose nanocrystals (CNCs) plasticized with 15 wt. % of acetyl(tributylcitrate) (ATBC) [32]. The incorporation of cellulose nanocrystals (CNCs) permitted the thermal stability, oxygen barrier and stretchability of the PLA-PHB blends to be improved. Bonilla et al. [29] added chitosan (CH) to PLA and obtained less rigid and less stretchable films, with no modification of PLA thermal properties. Likewise, the incorporation of chitosan led to higher water vapor permeability than that of neat PLA films while providing antimicrobial activity against total aerobial and coliform bacteria. PLA-Poly(butylene succinate-*co*-adipate) (PBSA) and Poly(butylene adipate-*co*-terephthalate) (PBAT) were obtained by Pivsa-Art et al. [33]. From the morphological study, it was found that an 80:20 PLA-PBSA polymer blend shows a worthy distribution of components and improved mechanical properties after the addition of PBAT, as an interfacial agent, at 10–30 wt. %, the highest tensile strength (40.71 MPa) being reached with 20 wt. % PBAT. Qin et al. [34] also developed PLA blend films with PCL (30 wt. %) by casting and characterized their physicochemical properties, as well as their antimicrobial activity, when carrying cinnamaldehyde, in packaged button mushrooms (*Agaricus bisporus*). The CO_2_ level inside the PLA-PCL packaging films with cinnamaldehyde was lower than that inside the control samples and in the PLA-PCL packaged samples, but the O_2_ level was similar in all packaged samples. The films with 9 wt. % cinnamaldehyde were the most effective at reducing microbial counts and at preserving the color of mushrooms, while showing the highest water vapor permeability. PLA and Poly(butylene succinate) (PBS) based films containing two different plasticizers (Acetyl Tributyl Citrate (ATBC) and isosorbide diester (ISE)] at three different contents (15, 20 and 30 wt. %) were produced by extrusion method [35]. The study showed that the two selected plasticizers were not effective at plasticizing PBS, while 15% of ISE was a suitable agent for increasing deformability of PLA-PBS (80–20). Other authors studied the PLA-starch (PLA-S) blends. Indeed, starch is a good candidate to obtain PLA blend films since it is widely available and cheap, with complementary properties to PLA. Nevertheless, the non-compatibility of both polymers requires the use of different compatibilization strategies, as discussed in Section 3.

## 3. The Main Characteristics of Starch Materials

**Figure 4:**
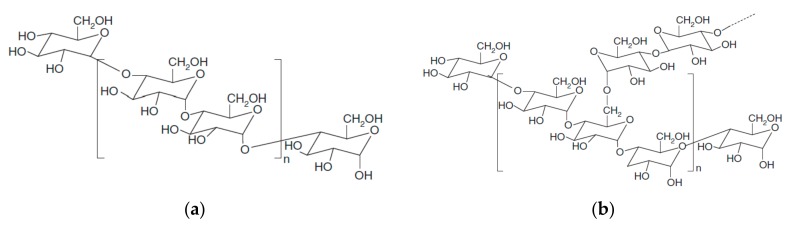
Amylose (**a**) and amylopectin (**b**) structures.

Starch is a plant polysaccharide consisting of several glucose units joined by glycosidic bonds. It is found in cereals (30–70% of the dry matter), tubers (60–90%) and legumes (25–50%) [51]. It is one of the most abundant biopolymers on earth, along with cellulose and chitin, being renewable, biodegradable and biocompatible. Native starch is composed of two main macromolecular components, which are amylose and amylopectin. Amylose is a nearly linear polymer of α-1,4 anhydroglucose units that has excellent film forming ability, rendering strong, isotropic, odorless, tasteless and colorless films [52]. Meanwhile, amylopectin is a highly branched polymer of short α-1,4 chains linked by α-1,6 glucosidic branching points occurring every 25–30 glucose units [53]. Figure 4 presents the amylose and amylopectin molecules and Table 2 shows the ratio of amylose/amylopectin of different starches, depending on their source.

Starch-based films have demonstrated several advantages, such as their extensibility and good oxygen barrier properties [55,56]. The oxygen permeability ranges between 0.4 × 10^−13^ and 2.5 × 10^−13^ cm^3^·m^−1^·s^−1^·Pa^−1^, depending on the film formulation and film-forming process [55,56,57]. They also exhibit similar physical characteristics to those of the conventional packaging plastics in terms of transparency, odor and taste [58]. Moreover, starch based films are reported to be nontoxic, contributing to their growing acceptance as a potential packaging alternative [59]. However, since it is highly hydrophilic, starch exhibits high water sensitivity and solubility and poor water vapor barrier capacity. The WVP of starch films can range between 1.2 × 10^−7^ and 8.3 × 10^−5^ g·m^−1^·s^−1^·Pa^−1^, depending on the film plasticization level or moisture content [60,61]. It is a low resistance material, with low tensile strength, depending on the moisture content [56,57,62,63]. Table 3 shows the values of the tensile properties of starch films reported by different authors, as affected by the source or glycerol content. A wide range in tensile strength (0.4–38 MPa) and elongation at break (1–129%) values can be found, depending on the level of plasticization of the polymer either by water or other components, such as glycerol. 

Starch-based films can be obtained by both thermo processing and casting techniques [56,66,67,68,69,70]. Nonetheless, films can only be obtained after the gelatinization of the native starch to irreversibly disrupt the granules. Gelatinization can be carried out with an excess of water (>90% w/w) [54] in the case of films obtained by casting or it can also be achieved at low moisture content during the thermal processing, by applying high-shear and high-pressure conditions; this is performed in the presence of plasticizers, such as water or glycerol, which tear down the starch granules, permitting a faster water transfer into the molecules and provoking the breakage of the amylopectin matrix, releasing the amylose.

Many applications have been described for starch. Indeed, it can be converted into chemicals (ethanol, acetone, and organic acids) and used in the production of synthetic polymers, it can be transformed into other biopolymers through fermentative processes or it can be hydrolyzed to provide monomers or oligomers. Likewise, it can be grafted with a variety of reagents to produce new derivative polymeric materials for different uses [54]. Starch has been used for pharmaceutical or cosmetic applications [71], as glue for paper and wood [72] or gum for the textile industry [73,74]. Likewise, native and modified starches play a more and more important role in the food industry, modifying the physical properties of food products, such as sauces, soups, or meat products [75], mainly resulting in textural changes, viscosity, adhesion, moisture retention, gel formation, and film formation [75]. Thus, in food applications, starch is used as a thickener, binder or setting agent in its granular form or as a sweetener or binder in its hydrolyzed form. Finally, in line with emerging research in bioplastics and because of its outstanding characteristics, starch has become one of the best options for food packaging applications. 

Thermoplastic starch, which is a rubbery material, is obtained by adding plasticizers to the native starch during its processing. Plasticizers can be fructose [76], sorbitol and maltitol [77], glycols [78], ethanolamine [79] or formamide [80]. Nevertheless, most studies report water and glycerol as the best plasticizing agents for starch. The amount of glycerol incorporated is generally between 25% [56,67] and 30% (w/w) [62,64]. Perry and Donald [81] showed that glycerol alone can completely gelatinize starch and induce an increase in the gelatinization temperature of approximately 60 °C, compared with the water-plasticized product. 

## 4. PLA-Starch Materials

To remedy the previously described disadvantages of both PLA and starch and to reduce the cost of the finished products, some alternatives have been figured out. Since both polymers exhibit opposite mechanical and barrier properties, their combination could lead to films with improved functional properties. However, since starch is highly hydrophilic and PLA is hydrophobic, they are thermodynamically immiscible. The biggest issue with mixing both polymers relies on the phase separation. To enhance their compatibility, either starch [82] or PLA [83] can be modified (e.g., with plasma treatment) in order to modulate their hydrophobicity. Nonetheless, the most widely investigated strategy is the use of PLA-starch blends. More recently, PLA-starch bilayer films have been studied to improve the functional properties of PLA-starch packaging materials.

### 4.1. Blend Films

Table 4 summarizes some of the most recent studies on PLA-starch blends, with or without compatibilizers, which are incorporated to enhance the interfacial interactions between the two polymers and Table 5 presents the tensile properties of blends (tensile strength and elongation at break values). Several studies have been carried out into the combination of both materials by blending them and adding different compounds. Several authors added methylenediphenyl diisocyanate (MDI) to the PLA-starch blend to enhance the compatibility of the two polymers [36,43,46]. They all found that this compound acted as a coupling agent, improving the interfacial interaction between the two materials, thus enhancing the mechanical properties of the PLA-starch blends. Wang et al. [46] reported a smoother microstructure and higher tensile strength (68 MPa) for the PLA-S blend (55:45) with 0.5 wt. % MDI. Acioli-Moura and Sun [36] reported a similar thermal decomposition profile of the blends, with or without MDI, although the compatibilizer led to a longer thermal endurance between 50 and 100 °C. MDI incorporation also gave an improved elongation at break from 2.7 to 4.2%. MDI is a small molecule, composed of isocyanate groups, which are highly reactive with both hydroxyl and carboxyl groups to form urethane linkages, thus being an effective compatibilizer for these two immiscible materials. Nevertheless, MDI is still recorded as a harmful substance by the EU Commission Communication 2008/C 34/01 [84] and cannot be used for food packaging applications.

Several studies were also carried out using maleic anhydride, grafted either on PLA [39,88] and/or starch [95], in order to improve the interfacial adhesion between the polymer phases. Orozco et al. [88] obtained PLA-g-MA copolymers using dicumyl peroxide (DCP) as an initiator of grafting and PLA-S copolymers were obtained by reactive blending, varying the starch composition from 0 to 60%. It was observed that MA had a plasticizing effect and enhanced the compatibility between PLA and starch, showing a stable and homogeneous interface with no stress fractures, holes nor cavities at the interface of the two polymers, as revealed by the SEM analysis. On the other hand, Zuo et al. [95] used the synthesis of MA esterified starches (1% MA) to blend it with PLA. The introduction of a hydrophobic ester bond in starch chains increased the polymer interfacial compatibility and led to an increase in the PLA-S water resistance and tensile properties. Some melt indexes showed that starch esterification also improved the melt flow properties of PLA-S composite material. MA was also grafted on both polymers by Hwang et al. [40], in a mixer in the presence of DCP in a one-step reactive compatibilization process. PLA-S (80:20) with 2.0 phr of MA and 0.1 phr of DCP showed the best tensile properties and the reactive compatibilization significantly reduced the combined molecular weight of the blend. Xiong Zhu et al. [93] also used tung oil anhydride (TOA), from 5 to 12 wt. %, as a bio-based reactive plasticizer for PLA-starch blends via the ready reaction of MA and observed a better compatibility between the two polymers, as demonstrated in the SEM micrographs and FTIR spectra, as well as a greater elongation at break (from 6 to 31%).

Le Bolay et al. [42] investigated the co-grinding of starch-PLA in a tumbling ball mill without adding any compatibilizer or plasticizer. The hydrophilic behavior of the blend decreased, as the interface between the matrix and the filler could be improved. Mechanical properties of the blends were improved, showing a higher ductility. Wokadala et al. [48] also studied the PLA-S blends without compatibilizers by using the butyl-etherification of waxy and high amylose starches to increase their hydrophobicity and compatibility with PLA. Although the polymer thermal stability decreased, the modified blend exhibited an improved mechanical performance, while SEM micrographs showed a more homogeneous structure with this starch modification. Additionally, this study demonstrated that the amylose/amylopectin content of starch plays an important role in the tensile properties of the starch-PLA blend films. At higher starch levels, composites with butyl-etherified high amylose starch gave a lower elongation at break and tensile strength, as compared to those with butyl-etherified waxy starch, due to the tendency of amylose to self-aggregate.

**Figure 5:**
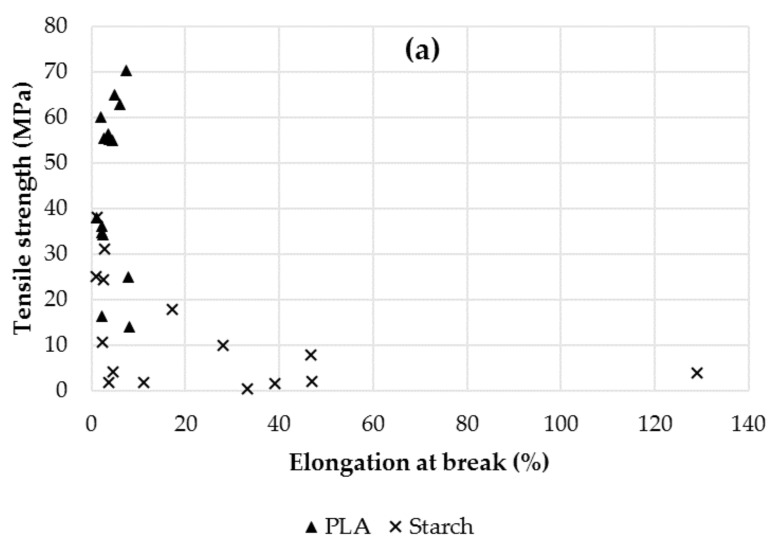

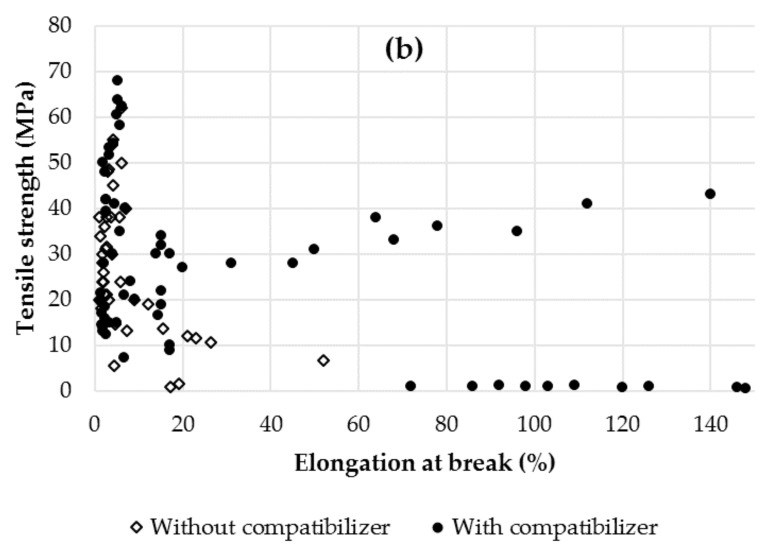
Map of tensile properties of neat PLA and starch (**a**); and of PLA-starch blends without and with compatibilizer (**b**).

As previously reported, the map of tensile properties of neat PLA and starch (Figure 5a), shows the poor resistance of starch and the poor extensibility of PLA. The map constitutes a plot of the pair variables TS vs. E% of different samples, reported by different authors, for a comparative purpose. On the blend map, with and without incorporation of a compatibilizer (Figure 5b), it is remarkable the general lack of effectiveness of the compatibilizers used. Only in a few cases, compatibilizers gave rise to an increase of the elongation at break (E), along with reasonable values of tensile strength (TS). Shirai et al. [61] obtained very high E values, in a range of 72–148%, using different adipate or citrate esters at 0.7 and 1 wt. %. However, all the films had very poor resistance, with TS values of less than 1 MPa. Xiong Zhu et al. [92] incorporated epoxidized soybean oil (ESO) in PLA-starch blends; with starch grafted with maleic anhydride to enhance its reactivity with ESO. Authors observed a marked increase in the elongation at break, from 6 to 112% and 140% with 9% and 13.3% of maleic anhydride, respectively, but the films were much more resistant that those of Shirai et al. [61] with a tensile strength of 43 MPa. The same authors [49] also used castor oil (5 wt. %) and hexamethylenediisocyanate (HDI)-graft starch, giving rise to extensible films with E values of 45 to 68%, compared to 6% without compatibilizers. Finally, in blends with 1.55% (w/w starch) of linoleic acid or tween 60, an emulsifier for the food industry, Yokesahachart and Yoksan [50] achieved to improve the elongation at break from 1 to 15%, without significantly changing the values of tensile strength (38 and 32–34 MPa, without and with compatibilizer, respectively).

Although relevant results showed that PLA-starch blending seems to be a good alternative method to enhance the film properties of net PLA or starch films, this requires the involvement of reactive processes, which, in turn, could lead to reactant residues in the films which must be tested as to their food safety. In contrast, the strategy of the bi or multilayer films did not require such great efforts to enhance the interfacial properties of the polymers since there is only contact at the layer interface. Moreover, in terms of barrier properties, a multilayer assembly exhibits more advantages, since the complementary barrier capacity of each polymer can yield a composite material with extremely high resistance to the mass transport of polar (water) and non-polar (gases) molecules, resulting in a very effective packaging material. Nevertheless, the good interfacial adhesion of the materials where there is contact between the layers must be ensured from the mechanical point of view. This aspect could be enhanced by using adequate adhesive materials, but studies on their migration are necessary, especially for food applications, where food safety can be compromised. Some studies carried out into biopolymer bilayer films are summarized in the next section.

### 4.2. Multilayer Films 

Very few multilayer film strategies have been studied, with the objective to optimize the functional properties of biodegradable packaging materials. Table 6 shows some works carried out into different materials assembled as bilayer films. Particularly for PLA-starch combinations in multilayer assemblies, Sanyang et al. [44] developed sugar palm starch (SPS) and PLA bilayer cast films (with different PLA contents, from 20 to 50 wt. %) and characterized their mechanical and thermal properties and water vapor barrier capacity. With a 50–50 PLA-starch ratio, the tensile strength increased but the elongation at break reduced drastically in comparison with neat starch films. However, the incorporation of a PLA layer significantly reduced the water vapour permeability, due to its hydrophobic resistance perpendicular to the transfer of water molecules. The film microstructure revealed a lack of strong interfacial adhesion between the starch and PLA cast layers. However, recently, Muller et al. [96] obtained very good adhesion between cast amorphous PLA and thermoprocessed cassava starch layers. The obtained bilayer films showed a great improvement in tensile and water vapor barrier properties despite the lower ratio of PLA sheet in the bilayer assembly (about 1/3 of the film thickness). The bilayer films maintained a high transparency and an oxygen permeability as low as that of net starch films. The PLA layer was used as a carrier of cinnamaldehyde to obtain antimicrobial bilayer films and the improvement in the barrier properties was maintained, with slightly lower mechanical resistance. Thermal analyses of bilayers revealed the diffusion of cinnamaldehyde or low molecular weight compounds from cast PLA layer to the starch adhered sheets, which contributed to plasticizing the amorphous regions. The results obtained offer an interesting option to obtain high barrier-highly resistant active films from thermoplastic starch and amorphous PLA, including cinnamaldehyde as active compound. 

Starch-PCL bilayer films were also obtained by Ortega et al. [70] by compression molding. Before compression, starch layers (neat starch and starch with 5% PCL) were plasticized by spraying aqueous solutions of ascorbic acid or potassium sorbate, for the purposes of favoring the starch-PCL adhesion. Authors showed that all bilayers enhanced barrier properties to water vapor and oxygen, as compared to neat S and PCL films, (up to 96% and 99% respectively). Potassium sorbate was the most effective at obtaining a better interfacial adhesion, thus improving the barrier and mechanical properties of the films and providing them with antimicrobial activity. Svagan et al. [97] managed to improve the oxygen permeability of PLA films by adding chitosan and montmorillonite (MMT) clay, which has been reported as non-cytotoxic [98]. Transmission electron microscopy revealed a well-ordered laminar structure in the deposited multilayer coatings, and the light transmittance of the films demonstrated the high optical clarity of the coated PLA. Bilayer films of the same materials could also be obtained with the objective of incorporating active compounds between the two layers. Requena et al. [99] added four active compounds (oregano and clove EO and their respective main compounds) at the interface of PHBV layers. Films with active agents were effective at controlling the bacterial growth of both *Escherichia coli* and *Listeria innocua*. Although tensile properties were not improved with respect to pure PHBV films, the active compounds yielded more transparent materials with improved WVP. The preparation and characterization of biodegradable bilayer films from isolated soy protein (SPI) and PLA were reported by González and Alvarez Igarzabal [15]. The tensile properties of the films were improved after the incorporation of the PLA layer, whereas the water vapor permeability decreased as compared to SPI films. SPI-PLA bilayers were also obtained and characterized by Rhim et al. [100], obtaining similar conclusions. Martucci and Ruseckaite [101], obtained biodegradable three-layer films, with PLA as the outer sheet and 30% glycerol-plasticized gelatin as the inner layer. This assembly exhibited significantly improved water vapor barrier capacity and mechanical resistance.

The multilayer strategy is often used in the packaging industry and more and more of the food available in the stores comes in high-tech plastic packaging, such as multilayer films, which ensures a longer-term preservation of the food than a monolayer structure. From this point of view, multilayer films based on PLA and starch offer a useful alternative for food packaging purposes, taking advantage of the complementary barrier properties of both materials, and supplying the films with the high mechanical resistance of PLA. In this sense, amorphous PLA has demonstrated good adhesion properties with thermoplastic starch while the layer combination may be adequate for different food contact purposes, depending on the water or fat content of the food and the potential interactions of the packaging layers with these food components. 

## 5. Conclusions

Combining PLA and starch to obtain biodegradable food packaging materials represents good alternative means to reduce plastic waste. The blending of these polymers in adequate proportions with the incorporation of some compatibilizers has yielded high-performance films which can meet several packaging requirements. For instance, different PLA-starch ratios (70–30 or 80–20) with epoxidized soybean oil and maleic anhydride, or castor oil and hexamethylenediisocyanate offered good mechanical resistance and flexibility and could be potential replacers of non-degradable petrochemical polymers on the basis of their functional properties, polymer availability, adequate food contact properties and competitive cost. On the other hand, PLA-starch multilayer films exhibited a very good barrier capacity for water vapor and gases, while demonstrating reinforced mechanical resistance with respect to net starch films. Likewise, this strategy offers different possibilities for food contact depending on the layer combination (according to whether the food is more or less hydrophilic). Moreover, PLA or starch sheets can act as carriers of active compounds to produce antimicrobial or antioxidant packaging. According to the chemical affinity of the active, their incorporation into the hydrophobic (PLA) or hydrophilic (starch) layers can be designed to produce the most effective material. 

## Figures and Tables

**Table 1:** Tensile properties of neat PLA films reported by different authors. Tensile strength (MPa) and Elongation at break (%).

PLA	Processing	TS (MPa)	E (%)	Ref.
120 kDa	Extrusion Compression moulding	55.1	3.8	[36]
1.1–1.7% D-content	Melt blending Compression moulding	16.4	2.1	[37]
99 kDa	Extrusion	34.1	2.5	[38]
2002D^®^ 4% D-content 235 kDa	Extrusion Injection moulding	60.0	2.0	[39]
4042D^®^ 6% D-content 130 kDa	Melt blending Compression moulding	56.3	3.6	[40]
2002D^®^ 4% D-content 235 kDa	Extrusion Blown moulding	34.6	2.1	[41]
12% D-content 68 kDa	Compression moulding	14.0	8.0	[42]
4042D^®^ 6% D-content 130 kDa	Melt blending Compression moulding	70.2	7.4	[43]
2000D^®^	Casting (Chloroform)	24.8	7.9	[44]
125 kDa	Extrusion Compression moulding	55.4	2.6	[45]
120 kDa	Compression moulding	62.7	6.1	[46]
12% D-content 160–220 kDa	Extrusion Compression moulding	36.0	2.1	[47]
2002D^®^ 4% D-content 235 kDa	Melt-blending Compression moulding	55.0	4.5	[48]
4032D^®^	Extrusion Injection moulding	65.0	5.0	[49]
4042D^®^ 6% D-content 130 kDa	Extrusion Injection moulding	38.0	1.0	[50]

^®^ Natureworks supplier.

**Table 2:** Amylose and amylopectin contents (%) in starch from different sources [54].

Starch	Amylose (%)	Amylopectin (%)
Wheat	30	70
Corn	28	72
Potatoe	20	80
Rice	20–30	80–70
Cassava	16	84

**Table 3:** Tensile properties of neat starch films reported by different authors. Tensile strength (MPa) and Elongation at break (%).

Starch	Glycerol Content (% w/w)	Process	TS (MPa)	E (%)	Ref.
Potato	from 20 to 40	Melt blending Compression moulding	1.8	3.6	[37]
Cassava	from 23 to 54	Melt blending Compression moulding	2.0	47.0	[43]
Corn			1.5	39.0	
Sugar Palm	30	Casting (Water)	7.7	46.7	[44]
Cassava	30	Extrusion Compression moulding	0.4	33.1	[45]
Corn	40	Extrusion Compression moulding	38.0	1.2	[46]
Cassava	33	Extrusion Injection moulding	25.0	1.0	[50]
Cassava	25	Casting (Water)	4.1	4.5	[56]
Cassava	from 15 to 30	Casting (Water)	3.8	129.0	[57]
Corn	30	Compression moulding	10.7	2.4	[64]
Corn	30	Casting (Water)	31.0	2.8	[60]
Corn	25	Casting (Water)	24.3	2.5	[55]
Corn	30	Compression moulding	10.0	28.0	[62]
Corn	25	Casting (Water)	17.9	17.1	[65]
Cassava	30	Compression moulding	1.7	11.0	[63]

**Table 4:** Examples of PLA-starch blends with or without incorporation of a compatibilizer.

PLA	Starch	Glycerol (% w/w S)	PLA-S Ratio	Compatibilizer	Compatibilizer Content	Processing	Ref.
Natureworks^®^	Wheat flour (65% starch)	20	25–75	Citric acid (CA)	from 0 to 20 wt %	Extrusion Injection moulding	[85]
120 kDa	Wheat	-	55–45	Methylene diphenyl diisocyanate (MDI)	0.05 wt %	Extrusion Compression moulding	[36]
1.1–1.7% D-content	Potato	from 20 to 40	40–60	Sodium montmorillonite (NaMMT)	0.5–1.0 phr (/dry S)	Melt blending Compression moulding	[37]
99 kDa	Maize	42	70–30 60–40 50–50	-	-	Extrusion	[38]
3052D^®^	Corn	33	80–20	Stearic acid (SA)	0.1 wt %	Melt blending Compression moulding	[86]
14 kDa	Corn	30	83–17 71–29 62–38 56–44	-	-	Melt blending Compression moulding	[87]
2002D^®^ 4% D-content 235 kDa	Wheat/Pea/Rice	from 30 to 39	83–27 57–43 40–60	a) Maleic anhydride (MA) b) 2,5-dimethyl-2,5-di-(*tert*-butylperoxy)-hexane	a) 2 wt % b) 0.1–0.25–0.5 wt %	Extrusion Injection moulding	[39]
4042D^®^ 6% D-content 130 kDa	Corn	-	90–10 80–20 70–30	a) Dicumyl peroxide (DCP) b) MA	a) 0.1 phr b) 2 phr	Melt blending Compression moulding	[40]
2002D^®^ 4% D-content 235 kDa	Cassava	-	90–10 70–30 50–50	Trimethoxy silane coupling agents: - 3-glycidoxypropyl trimethoxy silane (GP) - 3-aminopropyl trimethoxy silane (AP) - 3-chloropropyl trimethoxy silane (CP)	from 1 to 100% (w/w S)	Extrusion Blown moulding	[41]
12% D-content 68 kDa	Waxy maize (99% amylopectin)	-	80–20	-	-	Co-grinding Compression moulding	[42]
n.r.	Potato		85–15 75–25 65–35 50–50 40–60	MA	n.r.	Melt blending Compression moulding	[88]
4042D^®^ 6% D-content 130 kDa	Cassava/Corn	from 23 to 54	90–10 80–20 70–30 60–40	MDI PLA plasticizers: a) Propylene glycol (PG) b) Polyethylene glycol (PEG) 400 g·mol^−1^	MDI: 1.25% (w/w S) a) b) from 5 to 20 wt %	Melt blending Compression moulding	[43]
4% D-content 180 kDa	Corn	25	50–50	Anhydride functionalized polyester	1 wt %	Extrusion Injection moulding	[89]
3251D^®^	Cassava	33	6.3–93.7 6.0–94.0	Adipate or citrate esters	0.7–1 wt %	Blown extrusion	[61]
3251D^®^	Cassava	25–30	30–70	-	-	Extrusion Compression moulding	[90]
125 kDa	Cassava	30	80–20	CA SA	2% (w/w S)	Extrusion Compression moulding	[45]
120 kDa	Wheat	-	80–20 70–30 55–45 50–50 40–60	MDI	0.5 wt %	Compression moulding	[46]
12% D-content 160–220 kDa	Corn	40	50–50	CA	from 1 to 4% (w/w S)	Extrusion Compression moulding	[47]
12% D-content 160–220 kDa	Corn	from 10 to 40	50–50	Formamide	from 10 to 30% (w/w S)	Extrusion Compression moulding	[91]
2002D^®^ 4% D-content 235 kDa	Waxy maize (100% amylopectin)/High amylose maize (70% amylose)	-	40–60 30–70 20–80 10–90	-	-	Butyl-etherification of waxy and high amylose starch Melt-blending Compression moulding	[48]
4032D^®^	Corn	-	70–30 65–35	a) Hexamethylenediisocyanate (HDI) b) Castor oil	a) 5–8–11% (w/w S) b) 5 wt %	Extrusion Injection moulding	[49]
4032D^®^	Corn	-	90–10 80–20 70–30	a) MA b) Epoxidized soybean oil (ESO)	a) 4,3–9–13,3% (w/w S) b) 10 wt %	Extrusion Injection moulding	[92]
4032D^®^	Corn	-	70–30	Tung oil anhydride (TOA)	5–7–10–12 wt %	Extrusion Injection moulding	[93]
4032D^®^	Corn	-	70–30	a) Epoxidized itaconic acid (EIA) b) Bio-based ether epoxidized cardanol	20% (w/w S)	Extrusion Injection moulding	[94]
4042D^®^ 6% D-content 130 kDa	Cassava	33	70–30 50–50 30–70	Tween 60 Linoleic acid (LA) Zein	1.55% (w/w S)	Extrusion Injection moulding	[50]
Granular	Corn	50	60–40	MA	1% (w/w S)	Extrusion moulding	[95]

n.r.: non reported; ^®^ Natureworks supplier.

**Table 5:** Tensile properties of PLA-starch blends and bilayer films reported by different authors. Tensile strength (MPa) and Elongation at break (%).

PLA-S Ratio	Compatibilizer	Compatibilizer Content	Other Varying Factor (%)	TS (MPa)		E (%)		Ref.
				Without	With	Without	With	
0–70	-	-	-	6.7	-	52.0	-	[96]
50–50	-	-	-	13.7	-	15.5	-	[44]
40–60				12.1		21.0		
30–70				11.6		23.0		
20–80				10.7		26.4		
55–45	MDI	0.05 wt %	-	31.5	54.3	2.7	4.2	[36]
40–60	NaMMT	0.5 phr	-	5.6	7.3	4.3	6.7	[37]
70–30	-	-	-	14.5	-	4.5	-	[38]
60–40				13.3		7.3		
90–10	a) CP b) MA	a) 0.1 phr b) 2 phr	-	48.6	53.4	3.2	3.4	[40]
80–20				48.0	51.7	2.9	3.3	
70–30				35.9	41.9	2.1	2.6	
90–10	GP	-	-	23.9	19.6	2.0	1.4	[41]
	AP				21.6		1.3	
	CP				39.4		2.5	
80–20	-	-	-	19.0	-	12.0	-	[42]
90–10	MDI	1.25% w/w S	Cassava starch (25% Glycerol)	-	17.0	-	1.5	[43]
80–20					14.0		1.9	
70–30					13.0		1.8	
60–40					12.5		2.5	
90–10			Corn starch (25% Glycerol)		18.5		2.4	
80–20					16.0		2.3	
70–30					14.5		1.5	
60–40					15.0		3.2	
50–50	Anhydride functionalized polyester	1 wt %	-	18.0	28.0	1.5	2.0	[89]
93.7–6.3	Diethyl adipate	0.7 wt %	-	-	0.9	-	126.0	[61]
94–6		1 wt %			0.8		146.0	
93.7–6.3	Diisodecyl adipate	0.7 wt %			0.6		148.0	
94–6		1 wt %			0.7		120.0	
93.7–6.3	Acethyl triethyl citrate	0.7 wt %			1.2		109.0	
94–6		1 wt %			1.1		98.0	
93.7–6.3	Acethyl tributyl citrate	0.7 wt %			1.3		92.0	
94–6		1 wt %			1.1		86.0	
93.7–6.3	Tributyl citrate	0.7 wt %			0.9		72.0	
94–6		1 wt %			1.1		103.0	
30–70	-	-	25% Glycerol	1.7	-	19.2	-	[90]
			30% Glycerol	1.0		17.2		
80–20	CA	2% w/w S	-	-	16.5	-	14.5	[45]
	SA							
80–20	MDI	0.5 wt %	-	-	58.3	-	5.6	[46]
70–30					62.5		6.1	
55–45					68.1		5.1	
50–50					63.7		5.2	
40–60					60.6		4.9	
50–50	CA	2% w/w S	-	21.0	41.0	2.1	4.6	[47]
		4% w/w S			35.0		5.7	
	Formamide	30% w/w S		20.0	21.0	3.2	6.6	[91]
10–90	-	-	Waxy Starch	26.0	-	2.0	-	[48]
20–80				24.0		1.8		
30–70				20.0		1.4		
40–60				20.0		1.1		
10–90			Butyl-etherified Waxy Starch	54.0		4.0		
20–80				45.0		4.2		
30–70				38.0		5.5		
40–60				24.0		5.8		
10–90			High Amylose Starch	38.0		2.7		
20–80				31.0		2.5		
30–70				30.0		1.7		
40–60				28.0		1.8		
10–90			Butyl-etherified High Amylose Starch	55.0		4.1		
20–80				38.0		3.7		
30–70				30.0		3.8		
40–60				21.0		2.6		
70–30	HDI	5% w/w S	-	50.0	42.0	6.0	2.5	[49]
65–35	Castor Oil	5 wt%			40.0		7.0	
	HDI	5% w/w S			28.0		45.0	
		8% w/w S			31.0		50.0	
		11% w/w S			33.0		68.0	
90–10	ESO	10 wt %	-	62.0	38.0	6.0	64.0	[92]
80–20	MA	4.3% w/w S			36.0		78.0	
		9% w/w S			41.0		112.0	
		13.3% w/w S			43.0		140.0	
70–30					35.0		96.0	
70–30	TOA	5 wt %	-	40.0	30.0	7.0	17.0	[93]
		7 wt %			28.0		31.0	
		10 wt %			27.0		20.0	
		12 wt %			22.0		15.0	
70–30	EIA	20% w/w S	-	34.0	48.0	1.2	2.2	[94]
	Epicard				50.0		1.8	
30–70	Tween 60	1.55% w/w S	-	-	10.0	-	17.0	[50]
50–50					20.0		9.0	
70–30				38.0	34.0	1.0	15.0	
30–70	LA			-	9.0	-	17.0	
50–50					19.0		15.0	
70–30				38.0	32.0	1.0	15.0	
30–70	Zein			-	15.0	-	5.0	
50–50					24.0		8.0	
70–30				38.0	30.0	1.0	4.0	
60–40	MA	1% w/w S	-	20.0	30.0	9.0	14.0	[95]

**Table 6:** Recent studies on polymer bilayer films.

Polymers	Other Compounds (Content)	Polymer Ratio	Processing	Solvent for Casting (Polymer Content)	Ref.
1. PLA	1. PLA: Cinnamaldehyde (25%)	PLA-S	1. Casting 2. Compression moulding 1 + 2: Compression moulding	PLA: Ethyl acetate (10 wt %)	[96]
2. Cassava starch (S)	2. S: Glycerol (30%)	30–70			
1. Sugar palm starch (SPS)	SPS: Glycerol (30%)	SPS-PLA 50–50 60–40 70–30 80–20	Casting/Coating	1. SPS: Distilled water (8% w/w)	[44]
2. PLA				2. PLA: Chloroform (10% w/w)	
1. Corn starch	S: Glycerol (30%)	S-PCL Or S (5% PCL)-PCL	Melt blending Compression moulding	-	[70]
2. Polycaprolactone (PCL)					
1. Soy protein (SPI)	SPI: Glycerol (50%)	SPI-PLA 60–40^a^ 50–50^b^	Casting/Coating	1. SPI: Deionized water (0.75% w/w)	[15]
2. PLA				2. PLA: Chloroform (0.8^a^ Or 1.2^b^ % w/w)	
1. PLA	CH: Montmorillonite (MMT) (0.2 wt %)	-	1. PLA: Melt extrusion	CH solution: 0.235% (v/v) Acetic acid	[97]
2. Chitosan (CH)			2. Dipping of PLA film in CH/MMT solution	(0.2 wt %) with MMT	
1. CH	PAA: Ammonium peroxydisulfate ((NH_4_)_2_S_2_O_8_) & Gallium nitrate (Ga5NO_3_)_3_)	-	Electrosynthesis	-	[102]
2. Poly(acrylic) acid (PAA)					
1. Methylcellulose (MC) 2. Lipids: Paraffin oil/wax Or Hydrogenated Palm Oil (HPO) & Triolein	1. MC: PEG 400 (25%) 2. Lipids: Glycerol monostearate (GMS) (10%)	-	Layer chromatography spreading	MC: Water-ethyl alcohol (3:1 v/v)	[103]
1. FucoPol Polysaccharide	1. Fucopol: Citric acid (CA) (50%)	-	Casting/Coating	1. FucoPol: Distilled water (1.5% w/w)	[104]
2. CH	2. CH: Glycerol (50%) & CA (50%)			2. CH: 1 % w/w Acetic acid (1.5% w/w)	
1. Wheat gluten	1. Wheat gluten: Glycerol (20%) 2. PE: Ethylene/acrylic ester/maleic anhydride terpolymer Or ethylene/glycidyl methacrylate copolymer	-	1. Wheat gluten: Casting 2. Bilayer films: Thermocompression	Wheat gluten: Absolute ethanol (varying contents), acetic acid & water	[105]
2. Polyethylene (PE)					
1. PE (commercial)	PCL: Casein	-	PCL coating on PE film	PCL: Tetrahydrofuran (10%)	[106]
2. PCL	Or casein/ZnO nanoparticles (40%)				
Pol (3-hydroxybutyrate)-*co*-(3-hydroxyvalerate)] (PHBV)	Oregano EO Or Carvacrol Or Clove EO Or Eugenol (15% w/w)	50–50	Melt blending Compression moulding	-	[99]
